# Cytotoxicity of Medicinal Plant Species Used by Traditional Healers in Treating People Suffering From HIV/AIDS in Uganda

**DOI:** 10.3389/ftox.2022.832780

**Published:** 2022-05-02

**Authors:** Godwin Upoki Anywar, Esezah Kakudidi, Hannington Oryem-Origa, Andreas Schubert, Christian Jassoy

**Affiliations:** ^1^ Department of Plant Sciences, Microbiology & Biotechnology, College of Natural Sciences, Makerere University, Kampala, Uganda; ^2^ Fraunhofer Institute for Cell Therapy & Immunology (IZI), Leipzig, Germany; ^3^ Institute for Medical Microbiology & Virology, University Clinics & Faculty of Medicine, University of Leipzig, Leipzig, Germany

**Keywords:** cytotoxicity, glioblastoma cell-line, medicinal plants, herbalists, antiviral, HIV/AIDS, cytotoxic concentration, Uganda

## Abstract

**Introduction:** Many people living with HIV/AIDS (PLHIV) in Uganda widely use herbal medicines. However, their toxicity and safety have not been investigated. The use of these plants can potentially cause harmful effects to the health of patients. The purpose of this study was to determine the cytotoxicity of some commonly used medicinal plant species used by PLHIV.

**Methods:** The cytotoxicity of the plant extracts was determined with the AlamarBlue cell viability assay using the human glioblastoma cell line U87.CD4.CXCR4. The cells were treated with varying concentrations of extracts of *Warburgia ugandensis*, *Erythrina abyssinica*, *Cryptolepis sanguinolenta*, *Albizia coriaria*, *Psorospermum febrifugium*, *Gymnosporia senegalensis*, *Zanthoxylum chalybeum*, *Securidaca longipendunculata*, *Vachellia hockii*, *Gardenia ternifolia*, and *Bridelia micrantha* reconstituted with ethanol and dimethyl sulfoxide (DMSO). Using regression analysis, the half maximal cytotoxic concentration (CC_50_) of the plant extracts were calculated from exponential curve fits, since they provided the highest coefficient of determination, R^2^.

**Results:** The ethanol extracts of *W. ugandensis* (CC_50_ = 7.6 μg/ml) and *A. coriaria* (CC_50_ = 1.5 μg/ml) as well as the DMSO-reconstituted extracts of *W. ugandensis* (CC_50_ = 6.4 μg/ml) and *A. coriria* (CC_50_ = < 4 μg/ml) were highly cytotoxic. The cytotoxicity of *W. ugandensis* and *A. coriaria* compared well with the indigenous traditional knowledge of the toxic effects experienced when the plants were not used correctly. However, the cytotoxicity of most of the plant extracts (15/22) was low to moderate (CC_50_ = 21–200 μg/ml).

**Conclusion:** Most of the plant species tested in this study had low to moderate cytotoxicity against U87.CD4.CXCR4 cells, except *W. ugandensis* and *A. coriria* which were highly cytotoxic.

## Introduction

Traditional medicines are very popular and are widely used among people living with HIV/AIDS (PLHIV) (Ahmad, 2005; Mills et al., 2005; Anywar et al., 2020a, 2020b, 2020c). AIDS is the world’s commonest immunosuppressive disease. It is caused by the human immunodeficiency virus (Bedoya et al., 2002; Gea-Banacloche, 2006). There are more than 37.6 million PLHIV worldwide, with 61% of total in Sub Saharan Africa (UNAIDS, 2021). The HIV prevalence among adults in Uganda was 5.4%, though higher in females (6.8%) (UNAIDS, 2020). In Uganda, more than 90% of the population, especially in rural areas, rely on traditional herbal remedies to manage their daily health care needs (Kamatenesi-Mugisha and Oryem-Origa, 2007). This is largely attributed to the accessibility, affordability, and cultural appropriateness of the herbal medicine.

Traditional medicinal practitioners (TMP) in Uganda use over 235 medicinal plant species for treating PLHIV (Anywar et al., 2020a; Anywar et al., 2020b). We selected 11 popular medicinal plant species for cytotoxicity analysis from a previous ethnobotanical study conducted by Anywar et al. (2020a), on medicinal plant species used to treat PLHIV in Uganda. Most of the plants are boiled as decoctions or concoctions.

The use of traditional medicine has been commonly associated with severe side effects. In South Africa, for instance, high rates of dehydration, vomiting, diarrhoea, and altered mental status have been observed in patients who reported recent use of traditional medicines (Luyckx et al., 2004). Similarly, traditional herbal treatment has been associated with dysfunctions of the liver and kidneys resulting in high patient mortality (Luyckx et al., 2004). Acute renal failure is one of the most severe, but under-recognized, complications of traditional medicine use and yet it forms an important aetiology of renal diseases in routine clinical practice (Luyckx et al., 2004; Luyckx, 2012). About 35% of all cases of acute renal failure in Africa have been associated with the use of herbal remedies. Moreover, this figure is thought to be an underestimate because of the secrecy surrounding traditional health practices and the use of traditional remedies (Isnard Bagnis et al., 2004; Luyckx et al., 2004).

Contamination from ingestion of potentially toxic medicinal herbs, incorrect substitution of required medicine with toxic herbs, adulteration, herb-drug interactions, the use of suboptimal un-standardized herbal medicines and lack of regulations or their enforcement are some of the main challenges that plague the use of herbal medicines (Ernst, 2002; Liwa and Jaka, 2016). It is also known that the source, composition, and preparation of herbal remedies vary with prevailing local healing practices and level of knowledge and skill of the herbalist (Jha and Rathi, 2008; Anywar et al., 2020a).

Many herbs contain active compounds. Only a few of them have been evaluated for efficacy and safety, even when various reports have implicated many of them in toxicity (Jha & Rathi, 2008). The identities of toxic substances contained in African herbal medicines, their toxicology and pathogenesis are largely unknown (Luyckx et al., 2002; Anywar et al., 2021). This makes patients with pre-existing renal insufficiency or at risk of impairment more predisposed to kidney insults from such herbal remedies (Luyckx, 2012).

Cytotoxicity tests are useful to screen chemicals for their intrinsic and relative toxicities. This helps in determining the potential toxic or harmful effect of such compounds to human health that may occur inadvertently during use (Castaño and Gómez-Lechón, 2005; Schultz et al., 2020a). It is therefore important to establish their safety. The purpose of this study was to determine the cytotoxicity of some commonly used medicinal plant species treat people living with HIV/AIDS in Uganda.

## Methods

### Study Area

Plant extraction and preparation were done at the Fraunhofer Institute for Cell Therapy & Immunology (IZI), in Leipzig, Germany. The cytotoxicity assays were conducted at the Institute for *Medical Microbiology & Virology*, University Clinics & Faculty of Medicine, University of Leipzig, Leipzig, Germany, between June and December 2019.

### Collection of Plant Specimens

Eleven medicinal plant species were collected from different parts of Uganda between March and September 2017. The medicinal plant species administered were all used by herbalists to treat PLHIV in Uganda (Anywar et al., 2020a). Voucher specimens of the plant species were collected using standard procedures described in Martin (1995) and deposited in the Makerere University herbarium for identification. The identified specimens were classified using the Kew Medicinal Plant Names Services (MPNS), https://mpns.science.kew.org/mpns-portal/, on 9th December 2021. The plant families were checked against the Angiosperm Phylogeny Group IV. The plant species selected and their voucher numbers were: *Warburgia ugandensis* Sprague (AG383)*, Erythrina abyssinica* Lam. (AG608), *Cryptolepis sanguinolenta* (Lindl.) Schltr., (AG480), *Albizia coriaria* Welw. ex Oliv., (AG366), *Psorospermum febrifugium* Spach (AG483), *Gymnosporia senegalensis* (Lam.) Loes. (AG462), *Zanthoxylum chalybeum* Engl. (AG632), *Securidaca longipendunculata* Fresen. (AG363), *Vachellia hockii* (De Wild.) Seigler and Ebinger. (AG428), *Gardenia ternifolia* Schumach. and Thonn., (AG453) and *Bridelia micrantha* (Hochst.) Baill (AG452).

### Preparation of Plant Material

Except for *C. sanguinolenta* where the root or root bark was used, all the other plant extracts were obtained from the barks of the respective plant species. Fresh material from all plant species were chipped and air-dried for 3–4 weeks at room temperature in a well-ventilated room. The dried plant material was ground into fine powder using an electric mill. The plant materials were stored in air tight containers to protect them from oxidation following the World Health Organization guidelines on good agricultural and collection practices (GACP) for medicinal plants (WHO, 2003).

### Extraction of Plant Material

The plant material was extracted in 80% ethanol using the following procedure. About 1 g of pulverized plant material from each species was put in Eppendorf tubes. Then 10 ml of 80% ethanol was added to each of the tubes and vortexed to dissolve the plant material. The tubes were slowly and gently heated in a thermocycler to evaporate off the excess solvent at 35°C. All the extracts were made up to a concentration of 30 mg/ml by redissolving them in both DMSO and Ethanol. The Eppendorf tubes with the extracts were then labelled and stored in a refrigerator at 4°C for further tests.

### Cell Line

Cell viability was measured using the human glioblastoma cell line U87.CD4.CXCR4 obtained through the National Institute for Biological Standards and Control (NIBSC) Centre for AIDS reagents, United Kingdom. The U87.CD4.CXCR4 cell-line was selected for this study as the most appropriate because the plant extracts would ultimately have to be tested for antiviral activity against HIV-1. U87 glioma cell lines are stably transfected with CD4 and CCR5 or CXCR4, which they express (Bjorndal et al., 1997; Princen et al., 2004). Additionally, U87 cells have an infinite lifespan and are easy to handle (Allen et al., 2016). The U87 are a human primary glioblastoma cell line that is one of the most widely studied and commonly used cell lines (Westermark et al., 1973; Clark et al., 2010). The malignant glioma is an aggressive type of primary brain cancer in adults (Pollard et al., 2009) with a very poor survival rate of 36.5% after 1 year (Ostrom et al., 2014).

### Cell Preparation

The cells were prepared following the laboratory’s standard operating procedures and instructions from the manufactures. The cells were retrieved from storage under vapour phase of liquid nitrogen at −196°C. Then a 1 ml cell aliquot was quickly thawed in a water bath at 37°C. The cells were transferred to 9 ml warm Dulbecco’s Modified Eagles Medium (DMEM) supplemented with 10% foetal calf serum (FCS) and 1% antibiotics (100 U/ml Penicillin, 100 μg/ml Streptomycin (Gibco, Thermofisher, Life Technologies Ltd., United Kingdom) in a conical 15 ml tube and gently mixed. The cells were centrifuged at 1,200 rpm for 5 min to cause them to pellet. The medium was discarded and the cell pellet was gently resuspended in 10 ml of fresh warm medium.

The cells were divided into two T25 flasks containing 5 ml warm medium and incubated (Panasonic CO_2_ incubator, Model MCO-20AIC-PE, Japan) for 48 h at 37°C in a humidified atmosphere at 5% CO_2_, after which fresh medium was added. After the cells reached a confluence of 80–90%, they were split in a ratio of 1:4 to 1:10. The culture medium was removed and discarded. The cells were briefly rinsed with an equal volume of phosphate-buffered saline (PBS, pH 7.4) (Gibco/Invitrogen) which was discarded. The cells were then detached from the surface of the culture flask with 3 ml 0.25% (w/v) trypsin +0.53 mM EDTA, Gibco/Invitrogen, United States). The detached cells were observed under an inverted microscope. Then 10 ml of growth medium was added and cells were collected by gently pipetting. Different aliquots of the cell suspension were added to new culture vessels. The cells were incubated and harvested during the exponential growth phase. The viability of the cells was determined by trypan blue exclusion using a Neubauer counting chamber (Altman et al., 1993; Tran et al., 2011).

### Cytotoxicity Testing Using the AlamarBlue® Cell Viability Assay

The cytotoxicity of the plant extracts was determined using the AlamarBlue® (Thermo Scientific, United States) cell viability assay following the manufacturer’s manual. AlamarBlue is a water-soluble resazurin based reagent added to the cell culture that changes its colour upon chemical reduction by viable cells. Inhibition of cell metabolism, cell proliferation and destruction of cells by cytotoxic agents slows down the change of colour. Resazurin was used to quantitatively measure the viability of mammalian cell lines as a rapid, non-toxic, sensitive, and reliable growth indicator (O’Brien et al., 2000; Schoonen et al., 2012). Different concentrations of the plant extracts were tested in comparison to ethanol.

The plant extracts were prepared from a stock solution of 30 mg/ml in Eppendorf tubes. The extracts were pre-diluted 1:30 to a concentration of 1 mg/ml. Ethanol and DMSO were pre-diluted in medium to 6.2%. Then 100 µl of medium was added to each well of a 96-well flat-bottomed plate, followed by 100 µl of plant extract in the second row in duplicate. The medium and extracts were mixed in the wells followed by the transfer of 100 µl to the next row. The process was repeated with all diluted plant extracts. The carryover in the last row of 100 μl was discarded. Then 100 µl of cells was added to each well leading to plant extract concentrations starting with the undiluted row with the highest concentration of 250 μg/ml right through 125, 62.5, 31.3, 15.6, 8, and 4 μg/ml which was the lowest concentration. The 6.2% ethanol and DMSO were diluted in the same way. In the last pipetting phase, 20 μl of AlamarBlue reagent was added to each of the wells. The optical density (OD) was measured spectrophotometrically using an automatic plate reader (Tecan, Sunrise-Basic Tecan, Austria, GmbH, model 16039400) with a test wavelength of 570 nm and a reference wavelength of 600 nm. The readings were taken at 1, 3, 5, 24, and 50 h, respectively. Higher OD values indicated viable, metabolically active cells. Low OD values indicated cytotoxicity. The readings taken after 24 h of incubation were used to calculate the degree of metabolic activity in the presence of different concentrations of plant extract using the formula:

$$\text{D}\text{e}\text{g}\text{r}\text{e}\text{e}\;\text{o}\text{f}\;\text{m}\text{e}\text{t}\text{a}\text{b}\text{o}\text{l}\text{i}\text{c}\;\text{a}\text{c}\text{t}\text{i}\text{v}\text{i}\text{t}\text{y}=\frac{\text{M}\text{e}\text{a}\text{n}\;\text{O}\text{D}\;\text{w}\text{i}\text{t}\text{h}\;\text{s}\text{u}\text{b}\text{s}\text{tan}\text{c}\text{e}\;}{\text{M}\text{e}\text{a}\text{n}\;\text{O}\text{D}\;\text{w}\text{i}\text{t}\text{h}\text{o}\text{u}\text{t}\;\text{s}\text{u}\text{s}\text{b}\text{s}\text{tan}\text{c}\text{e}\;}$$

### Data Analysis

Regression analysis was performed using Microsoft Excel 2011. The 50% cytotoxicity values (CC_50_) of the plant extracts and the coefficient of determination R^2^ were calculated. The CC_50_ values represent the concentration that reduced the chemical reduction potential of the cell culture by 50%. The coefficient of determination R^2^ indicates the goodness of fit of the measured values and the regression curve. To determine CC_50_ values, a best curve fit analysis was performed with linear, logarithmic, exponential, and polynomic curve equations offered by the Microsoft Excel computer spreadsheet program. Exponential curves provided the highest coefficient of determination, R^2^, in most cases. Therefore, exponential curve equations were used to calculate the CC_50_ values.

## Results

The National Cancer Institute (NCI) classified cytotoxic compounds as: 1) highly active if IC_50_ ≤ 20 μg/ml, 2) moderately active if IC_50_ = 21–200 μg/ml, 3) weakly active if IC_50_ = 201–500 μg/ml and inactive if IC_50_ = > 501 μg/ml (Geran, 1972). Using the NCI system with a slight modification to replace activity with cytotoxicity, we categorized both the ethanol and DMSO-reconstituted extracts of *A. coriaria* and *W. ugandensis* as highly cytotoxic. Generally, there was a dose dependent increase in cytotoxicity with all the plant extracts tested.

Extracts from *V. hockii*, *B. micrantha*, *E. abyssinica*, *G. ternifolia* Subsp. *jovis-tonantis*, *P. febrifugium*, *G. senegalensis*, *C. sanguinolenta* (DMSO extract), *S. longipendunculata* (DMSO extract), and *Z. chalybeaum* (EtOH extract) were moderately cytotoxic. Extracts from *C. sanguinolenta* (EtOH extract), *S. longipendunculata* (EtOH extract), and *Z. chalybeaum* (DMSO extract) were weakly cytotoxic. Both ethanol and DMSO did affect cell growth by less than 10% at a concentration of 0.83 %v/v which was the maximum concentration of extractant present in the tested plant extracts (Table 1).

**TABLE 1:** Cytotoxicity (CC_50_) of the ethanol & DMSO extracts of the different plant species tested.

Plant species	CC_50_ (µg/ml)	CC_50_ (µg/ml)
	EtOH extract	DMSO extract
1. *A. coriaria*	6.4	14.9
2. *B. micrantha*	96.8	96.7
3. *C. sanguinolenta*	246.1	118.2
4. *E. abyssinica*	65.9	72.9
5. *G. ternifolia Subsp. jovis-tonantis*	53.0	76.2
6. *G. senegalensis*	70.2	81.8
7. *P. febrifugium*	112.0	128.2
8. *S. longipendunculata*	>250	98.3
9. *V. hockii*	100.3	89.0
10. *W. ugandensis*	7.2	2.0
11. *Z. chalybeaum*	>250	>250
Controls (Ethanol, DMSO)	4.9%	4.0%

## Discussion

The aim of the examination was to determine the toxicity of ethanol and DMSO extracts of selected medicinal plants in a cell culture model. The glioblastoma cell line U87 was chosen for this analysis because the existence of a U87 model for examining the inhibition of HIV infection *in vitro* (Bjorndal et al., 1997; Princen et al., 2004). The data on inhibition of HIV is still under review. Several methods are in use to examine the toxicity of medicinal compounds in cell culture (Cost et al., 2002; Auld et al., 2008; Wang et al., 2014). Repeated measurements of cytotoxic effects at several time points were done. Above pH 6.5, resazurin stains the cell culture medium purple. Upon chemical reduction, the colour of the cell culture medium changes to pink. Resazurin reduction occurs in viable, untreated cells. The colour of the cell cultures were measured spectrophotometrically. High absorbance of light (optical density) at 570 nm (pink) indicated viable cells whereas low optical density values (purple) indicated cytotoxicity. We monitored the metabolism of the cells for 5–50 h. The 24 h values were chosen for the calculation of the CC_50_, because untreated cells reached maximum pink colour intensity at this time point.

Cytotoxicity was examined with twofold dilutions of the extracts. A concentration range between 4 and 250 μg/ml was optimal for most of the extracts. The coefficients of determination were greater than 0.7 in all but one instance indicating that the calculated CC_50_ values were fairly precise. At 250 μg/ml diluted extracts contained 0.83% EtOH and DMSO. At this concentration, EtOH and DMSO alone showed less than 10% cytotoxicity indicating that cytotoxicity of the extractants was negligeable (Table 1 and Supplementary Figure S1).

Herbalists generally prepare the medicinal plant extracts using water. However, in our experiments, ethanol and DMSO were used for the extraction. The ethanol extracts were generally less cytotoxic than the DMSO extracts. A compound is regarded to be cytotoxic if it prevents cellular attachment, causes dramatic morphological changes, adversely affects replication rate, or leads to a reduction in overall viability (Horvath, 1980). It is worth noting that the expression of these effects depends on the length of time of exposure of the cells to the particular compounds, mechanism of cytotoxicity and the kind of compound under test (Riss and Moravec, 2004; Di Nunzio et al., 2017).

Other studies have also shown *W. ugandensis* and *A. coriaria*, the most highly cytotoxic plant species in our study to be cytotoxic in other cell lines. Mwitari et al. (2013) showed the ethanol extracts of *W. ugandensis* were cytotoxic for intestinal epithelial cells IEC-6, (IC_50_ < 50 μg/ml). *W. ugandensis* contains cytotoxic sesquiterpenes called muzigadials (Olila et al., 2001) and causes toxic side effects when the recommended dosage is exceeded as shown in Table 2. However, water extracts of *W. ugandensis* extracts were considered nontoxic in Vero E6 cells (CC_50_ > 250 μg/ml) and in BALB/c mice (LD_50_ > 5000 mg/kg body weight, Karani et al., 2013). Both the DMSO-reconstituted and ethanol extracts of *A. coriaria* from this study were highly cytotoxic. Kigondu et al. (2009) showed that the methanol and aqueous extracts of *A. coriaria* had very low cytotoxicity (CC_50_ > 500 μg/ml) against human embryonic lung fibroblast cells (HELF). The methanol, ethanol, ethyl acetate and diethyl ether extracts of *A. coriaria* were not toxic for the human keratinocyte cell line HaCaT (IC_50_ > 512) (Schultz et al., 2020b). *Albizia* spp. contains a toxic compound, 4-methoxypyridoxine that is a vitamin B6 antagonist (Botha and Penrith, 2008). *A. coriaria* and *W. ugandensis* are widely used in traditional herbal preparations as well as commercial herbal preparations in Uganda (Anywar et al., 2020a; Kaggwa et al., 2022).

**TABLE 2:** A comparison of cytotoxicity and medical indication, use and traditional knowledge on toxicity of *A. coriaca* and *W. ugandensis*

Additional information	Plant extract	
	*A. coriaca*	*W. Ugandensis*
CC_50_ Ethanol extract	6.4 μg/ml	7.2 μg/ml
CC_50_ DMSO extract	14.9 μg/ml	2.0 μg/ml
Preparations in use	Decoctions, herbal baths or ointments	Decoctions, herbal baths or ointments
Special instructions for use	Prolonged boiling (up to 6 h & used in combination with other herbs for in small amounts	Prolonged boiling (up to 6 h & used in combination with other herbs for in small amounts
Medical indications	Cancer, heart disease, allergy, nausea, headaches & mental illness, diarrhoea, cough, TB, anaemia, fatigue, stomach & skin lesions (G. Anywar et al., 2020a)	Stomach ache, sore throat, ulcers, fatigue, fever, blood infections cough/TB/asthma, diarrhoea, allergies, syphilis, skin lesions/rash (G. Anywar et al., 2020a)
Contraindications	Pregnancy, very weak patients	Pregnancy, very weak patients
Known side effects	Vomiting, dizziness, weakness	Vomiting, dizziness, weakness, ulcers

The contraindications only apply for the decoctions which are administered orally.

*B. micrantha* showed moderate cytotoxicity in our study. In other studies, *B. micrantha* was cytotoxic to HeLa cells (IC_50_ = 8.9 μg/ml), human breast cells (MCF-12A, IC_50_ = 24.2 μg/ml) (Steenkamp et al., 2009), CCRF-CEM leukaemia cells (IC_50_ = 9.43 μg/ml and 23.5 μg/ml) for leaf and bark extract in dichloromethane and methanol, respectively (Omosa et al., 2016). *B. micrantha* has also been shown to be toxic to brine shrimp (50% lethal concentration, LC_50_ = 77 μg/m) for the water stem bark extract (Osebe et al., 2016) but mildly toxic for the ethanol root extract (LC_50_ = 30 μg/ml) (Moshi et al., 2010).

The ethanol extracts of *P. febrifugium* exhibited significant *in vivo* cytotoxicity against P-388 lymphocytic leukaemia cells (3 PS) in mice (Abou-shoer et al., 1989). Psorospermin is a cytotoxic dihydrofuranoxanthone first isolated from the roots of *P. febrifugum* (Gebre-Mariam et al., 2006). Psorospermin showed significant activity against human colon adenocarcinoma (HT-29), human lung carcinoma (A-549), and human breast adenocarcinoma (MCF-7) cell lines (Cassady et al., 1990).

The ethanol root extract of *S. longipendunculata* significantly inhibited proliferation of U87 cells (IC_50_ = 20.54 μg/ml) (Ngulde et al., 2019). The chloroform root extract of *S. longipedunculata* was preferentially cytotoxic to PANC-1 human pancreatic cancer cells. The aqueous root bark extract was toxic to Ehrlich ascites tumour cells with a cytotoxicity of 82.5% at 1000 μg/ml, and an IC_50_ of 67 μg/ml (Lawal et al., 2012). The methanol root extract of *S. longipendunculata* is relatively toxic to brine shrimp with a LC_50_ of 77.1 μg/ml (Moshi et al., 2006), LC_50_ of 74.18 μg/ml (Adiele et al., 2013). The extract was also toxic in albino mice (LD_50_ = 1.74 g/kg and 0.02 g/kg) when administered orally and intraperitoneally, respectively (Adeyemi et al., 2010). Toxic saponins and methyl salicylate, a suspected nephrotoxin from *S. longipendunculata* can cause renal damage (Colson and De Broe, 2005). Xanthones from *S. longipedunculata* exhibited potent and selective cytotoxicity against PANC-1 human pancreatic cancer cells (Dibwe et al., 2013). The aqueous root extract of *C. sanguinolenta* exhibited potent toxicity to a variety of mammalian cells *in vitro* including the Chinese hamster lung fibroblast cell line V79-MZ) (Ansah and Gooderham, 2002). *C. sanguinolenta* contains the alkaloid cryptolepine and isocryptolepine (Grellier et al., 1996; Bonjean et al., 1998). Cryptolepine was cytotoxic to B16 melanoma cells (IC_50_ = 0.3 μg/ml) (Bonjean et al., 1998). Skilled herbalists normally administer known toxic medicinal plants albeit in low doses and in polyherbal preparations (Anywar et al., 2020a). This can potentially neutralize the toxicity of some medicinal plant compounds. However, polyherbal preparations can also have unpredictable and complicated effects arising from the various interactions that can occur among individual components (Che et al., 2013; Di Nunzio et al., 2017). The cytotoxicity data on the two highly cytotoxic plants: *W. ugandensis* and *A. coriaria* corresponds to the traditional knowledge of herbalists from our earlier study (Anywar et al., 2020a) about the toxic side effects experienced with the incorrect use of these plants. Although single species plant extracts were tested, it is common for the herbalists to use mixtures of multiple plant species for treatment. Therefore, the corresponding cytotoxicities of standard plant extract mixtures need to be investigated.

## Conclusion

Most of the plant species extracts tested in this study had low to moderate cytotoxicities against U87.CD4.CXCR4 cells *in vitro*, except for *W. ugandensis* and *A. coriria extracts* which were both highly cytotoxic. The high *in vitro* cytotoxicity levels of *W. ugandensis* and *A. coriria* correspond well with the toxic effects experienced by people who incorrectly used these herbs as reported by herbalists. Therefore, the correct use of herbal medicines is important in mitigating toxic side effects.

## Data Availability

The datasets presented in this study can be found in online repositories. The names of the repository/repositories and accession number(s) can be found below: https://data.mendeley.com/datasets/25kdprt3ch/2, doi: 10.17632/25kdprt3ch.2.
