# Supplementary figures and images for: Cytotoxicity of Medicinal Plant Species Used by Traditional Healers in Treating People Suffering From HIV/AIDS in Uganda
Source: Front Toxicol. 2022 May 2;4:832780. doi: 10.3389/ftox.2022.832780 (PMC9108544; doi:10.3389/ftox.2022.832780)

Supplementary Figure 1A, ethanol extracts

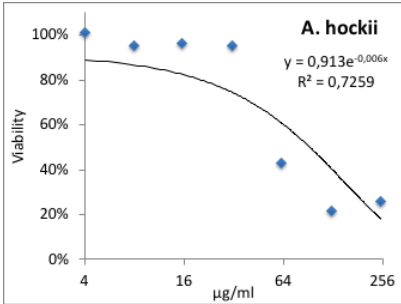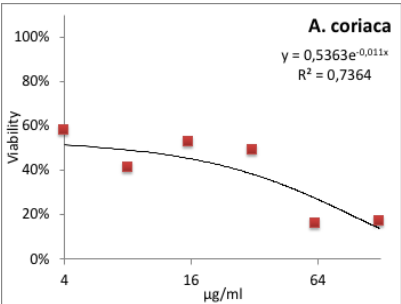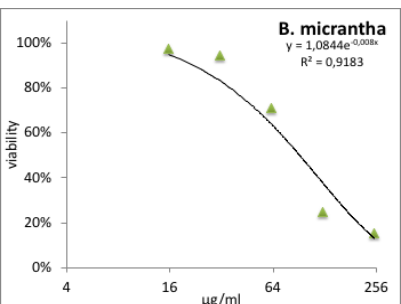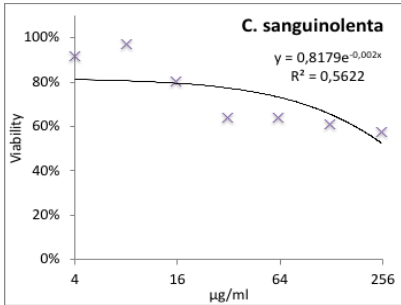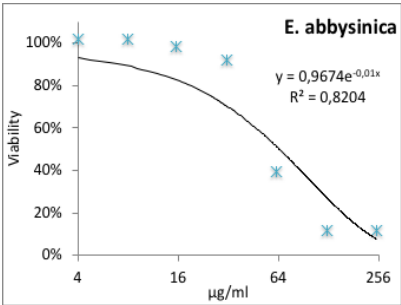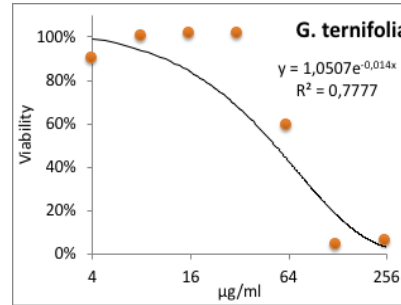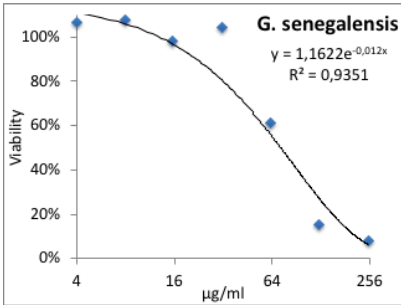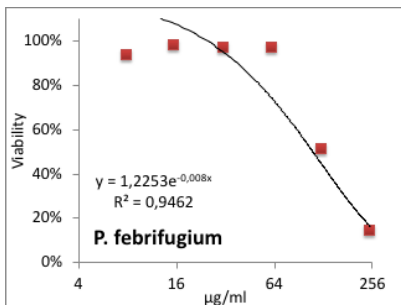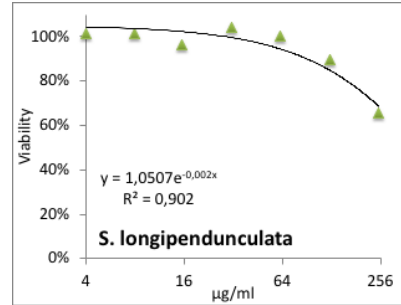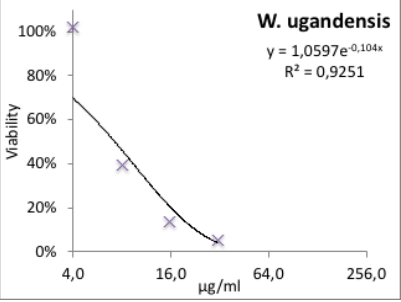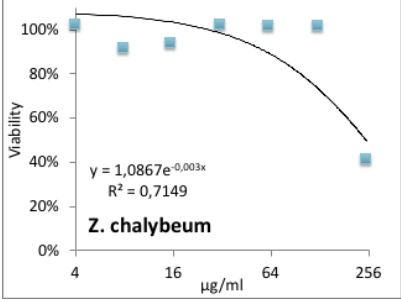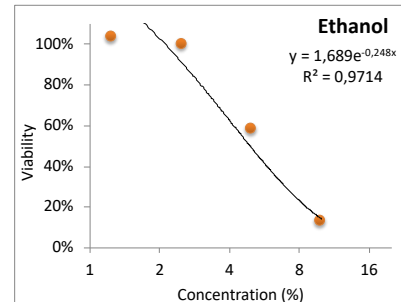

Supplementary Figure 1B, DMSO extracts

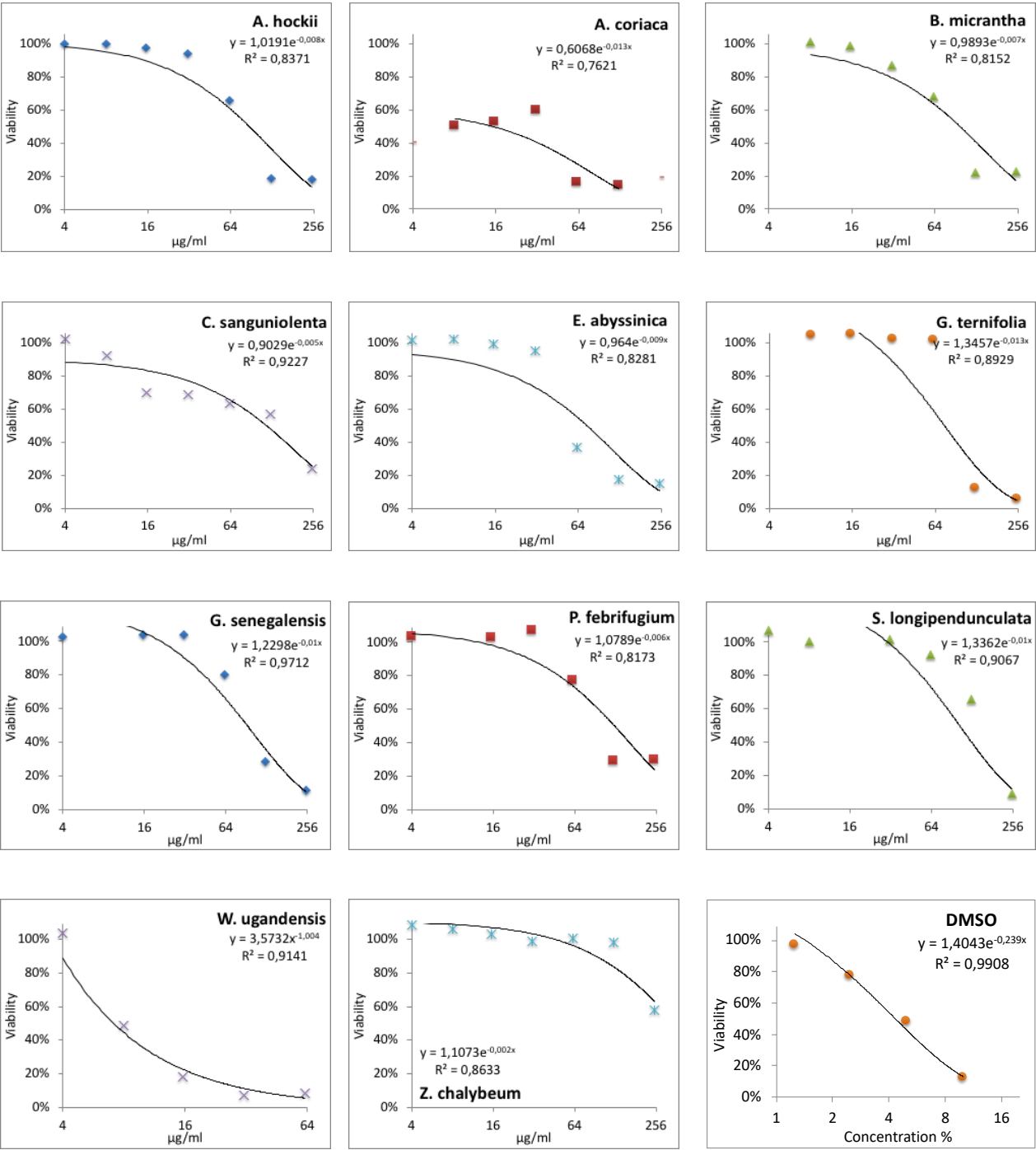

Supplement: Supplementary file 1 [file Presentation1.pdf]
